# Supplementary material for: Phage libraries screening on P53: Yield improvement by zinc and a new parasites-integrating analysis
Source: PLoS One. 2024 Oct 3;19(10):e0297338. doi: 10.1371/journal.pone.0297338 (PMC11449285; doi:10.1371/journal.pone.0297338)
Supplement: S1 Table — a) all peptides except those of R set. b) R set representatives for 2LY4B docking. And c) R set representatives for 3Q01 docking. (Pep: peptides). (PDF) [file pone.0297338.s001.pdf]

Peptide	Sequence	E (2LY4B)	E (3Q01)
7.1	HTWLRSA	-5,4	-5,8
7.2	LHNSLPA	-4,7	-4,7
7.3	NPNSAQG	-3,9	-5,7
7.4	ATHQTLR	-3,7	-5,6
7Z1	WSWPRFL	-4,9	-5,9
7Z2	MQAPSPM	-4,1	-5,8
7Z3	AAAFTQS	-4,8	-5,7
7Z4	GTEPPAM	-4,3	-5,5
12.1	NHMNQISFPSRP	-4,3	-5,6
12.2	ARSPCQVQSRTS	-4,1	-4,1
12.3	NNLAFYHTFISP	-4,6	-6,8
12.4	APSPFQVQSRTS	-5,9	-6,4
12.5	NYPSSSVPHAPQ	-4,5	-6,9
12.6	YSTHDNARPWLL	-5	-5,3
12Z1	SHVPLARWSVIT	-4,8	-6
12Z2	HDHLIPFYWADL	-5,2	-6
12Z3	STLVFPAHTRDY	-4,2	-4,9
12Z4	TYLLPHSYPWYG	-4,6	-6,8
12Z5	TATLDMPLSLPS	-3,9	-6,2
12Z6	WMDSYMSQHDWP	-4,3	-5
PD.1	GANMKYA	-3,7	-5,4
PD.2	GLTATNM	-4,7	-5
PD.3	GFTATNM	-5,1	-5,8
PD.4	NDAEMPT	-2,8	-5,6
PD.5	ETTHARA	-3,9	-6,4
PD.6	GLDCYKQ	-3,7	-5,9
PD.7	STQARTP	-4,9	-6,1
SR12.1	HLAQTASPPAAP	-4,9	-6,3
SR12.2	APLYSPSHLATS	-5,7	-5,9

a

Pep	Sequence	E (2LY4B)
R0	VGVRIPL	-4,2
R1	NGVEIPP	-5
R3	PFNEPHP	-5,4
R4	PINEPHP	-5,5
R6	SFNEPHP	-4,8
R7	SINEPHP	-4,7

b

Pep	Sequence	E (3Q01)
R0	VGVRIPL	-6,2
R1	NGVEIPP	-6,8
R3	PFNEPHP	-6,9
R4	PINEPHP	-6,7
R2	PFNEPHL	-7
R5	PKNEPHP	-7,1
R8	VGVGIPP	-5,9
R9	PGVGIPL	-5,9
R10	IRVGIPL	-6,2
R11	LFNERHP	-6
R12	PINEPHL	-6,7
R13	AFNEPHP	-5,7
R14	AINEPHP	-5,9
R15	AINEPHL	-6
R16	AFHEPHP	-7,4
R17	AIHEPHP	-5,9

c

S1 Table. Energy of docking. a) all peptides except those of R set. b) R set representatives for 2LY4B docking. And c) R set representatives for 3Q01 docking. (Pep: peptides)
