# Supplementary material for: Phage libraries screening on P53: Yield improvement by zinc and a new parasites-integrating analysis
Source: PLoS One. 2024 Oct 3;19(10):e0297338. doi: 10.1371/journal.pone.0297338 (PMC11449285; doi:10.1371/journal.pone.0297338)
Supplement: S1 Fig — Peptides are: 7.1–7.4 (non zinc) and 7Z1-7Z4 (with zinc). (PDF) [file pone.0297338.s002.pdf]

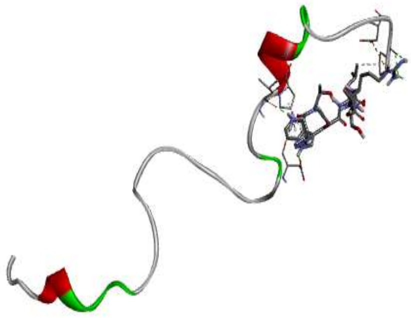

7.1: HTWLRSA

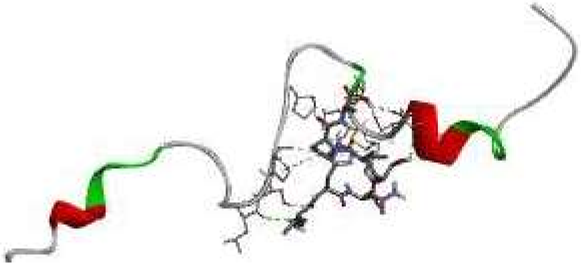

7.2 : LHNSLPA

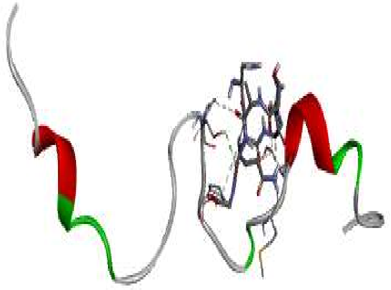

7.3: NPNSAQG

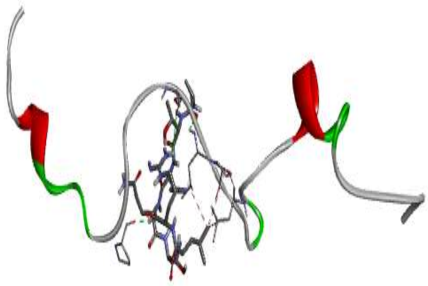

7.4 : ATHQTLR

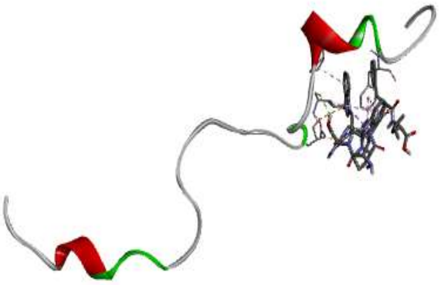

7Z1 : WSWPRFL

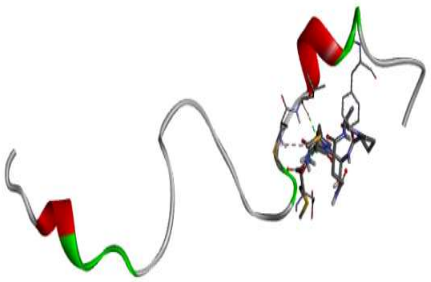

7Z2 : MQAPSPM

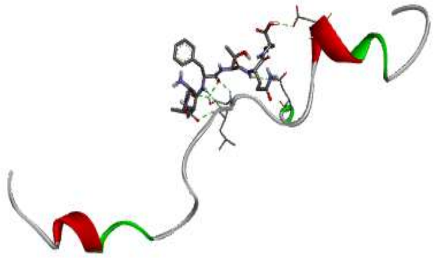

7Z3 : AAAFTQS

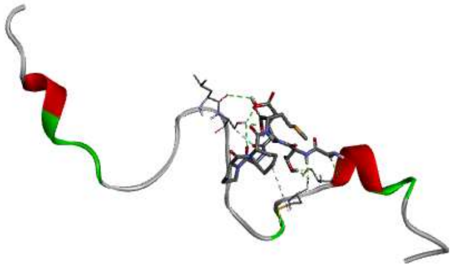

7Z4 : GTEPPAM

S1 Fig. Docking structures of 7-mer set with 2LY4.B. Peptides are : 7.1-7.4 (non zinc) and 7Z1-7Z4 (with zinc).
