# Supplementary material for: Phage libraries screening on P53: Yield improvement by zinc and a new parasites-integrating analysis
Source: PLoS One. 2024 Oct 3;19(10):e0297338. doi: 10.1371/journal.pone.0297338 (PMC11449285; doi:10.1371/journal.pone.0297338)
Supplement: S3 Fig — Peptides are 12Z1-12Z6. (PDF) [file pone.0297338.s004.pdf]

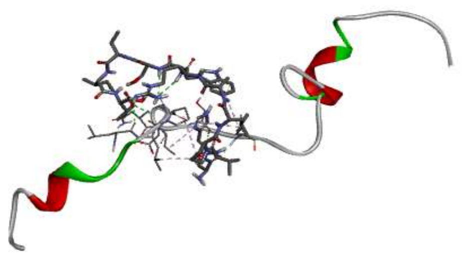

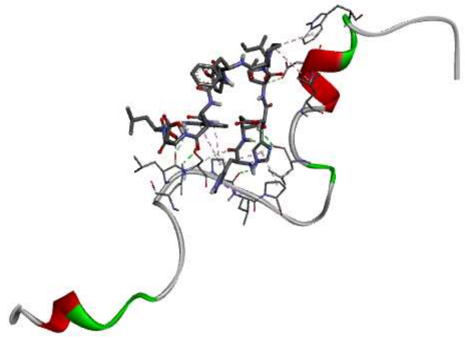

12Z1:SHVPLARWSVIT 12Z2: HDHLIPFYWADL

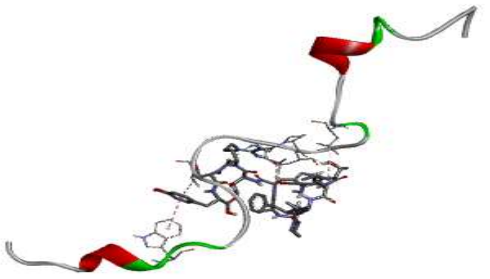

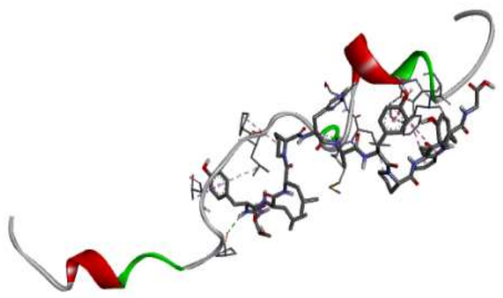

12Z3:STLVFPAHTRDY 12Z4:TYLLPHSYPWYG

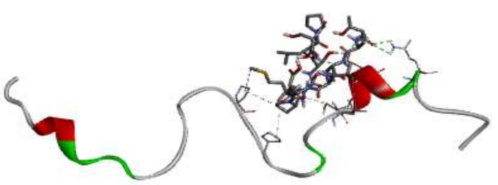

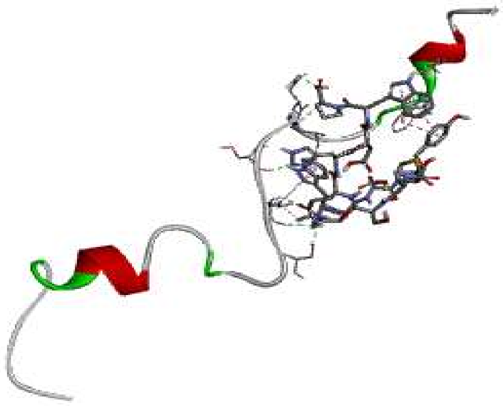

12Z5: TATLDMPLSLPS 12Z6:WMDSYMSQHDWP

S3 Fig. Docking structures of 12-mer *"With Zinc"* set with 2LY4.B. Peptides are 12Z1-12Z6.
