# Supplementary material for: Phage libraries screening on P53: Yield improvement by zinc and a new parasites-integrating analysis
Source: PLoS One. 2024 Oct 3;19(10):e0297338. doi: 10.1371/journal.pone.0297338 (PMC11449285; doi:10.1371/journal.pone.0297338)
Supplement: S4 Fig — (PDF) [file pone.0297338.s005.pdf]

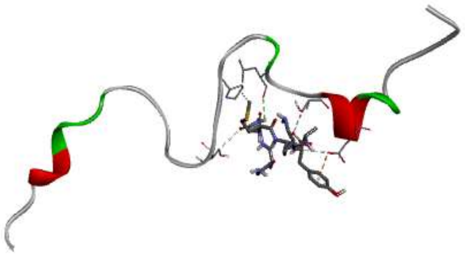

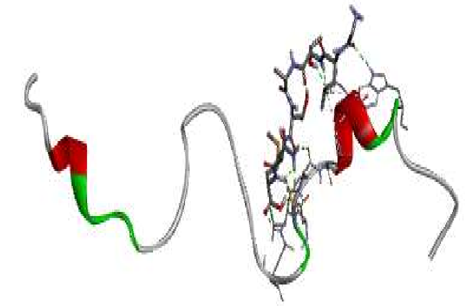

PD1: GANMKYA

PD2: GLTATNM

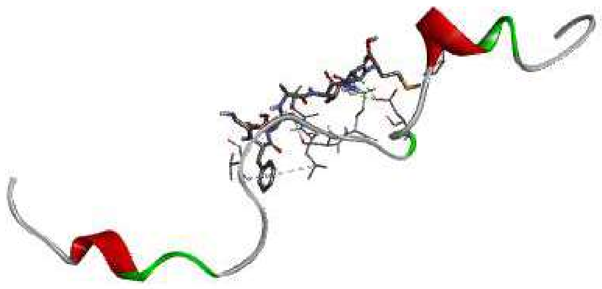

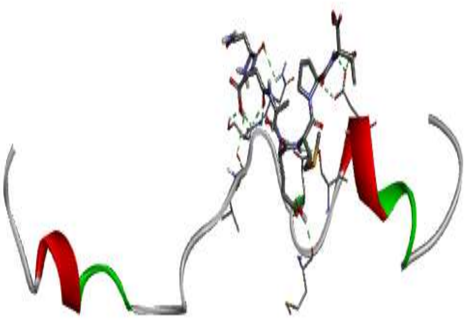

PD3: GFTATNM

PD4: NDAEMPT

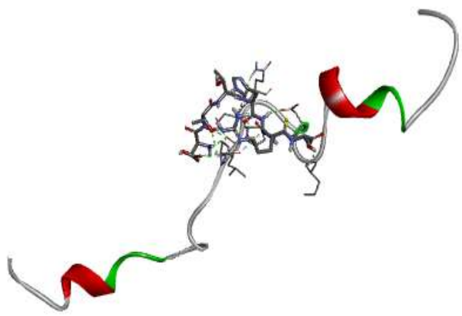

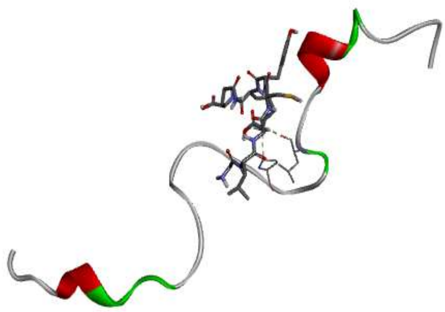

PD5: ETTHARA

PD6: GLDCYKQ

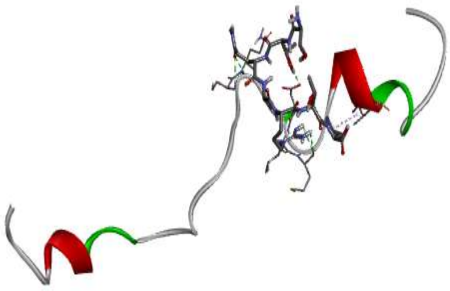

PD7: STQARTP

S4 Fig. Docking structures of PD74 set with 2LY4.B. Peptides are PD1-PD7.
