# Supplementary material for: Phage libraries screening on P53: Yield improvement by zinc and a new parasites-integrating analysis
Source: PLoS One. 2024 Oct 3;19(10):e0297338. doi: 10.1371/journal.pone.0297338 (PMC11449285; doi:10.1371/journal.pone.0297338)
Supplement: S5 Fig — a. SR50: SR12.1 and SR12.2. b. Representatives of Redundant (R) peptides: R0-R3, R6 and R7. (PDF) [file pone.0297338.s006.pdf]

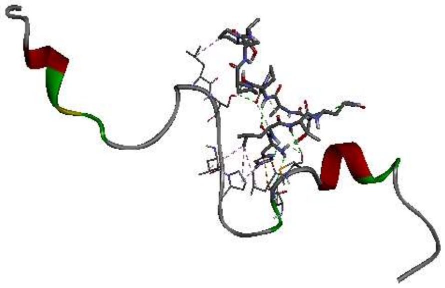

SR12.1 : HLAQTASPPAAP

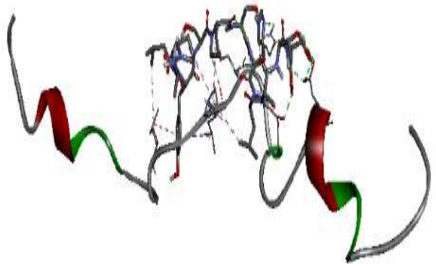

SR12.2 : APLYSPSHLATS

a. SR50 peptides.

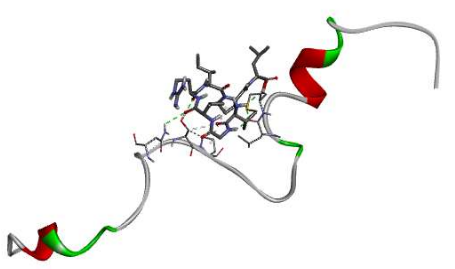

R0 : VGVRIPL

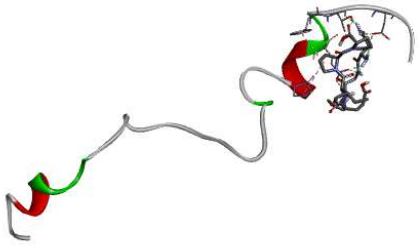

R1 : NGVEIPP

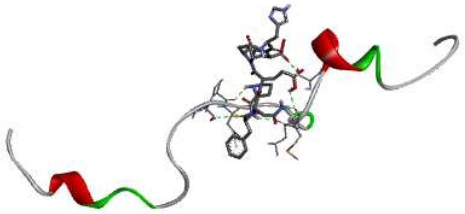

R2 : PFNEPHP

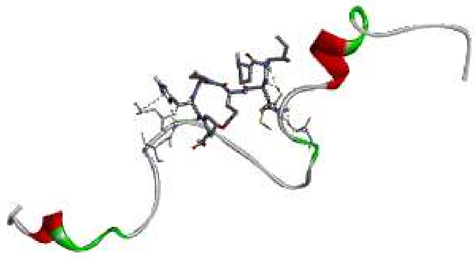

R3 : PINEPHP

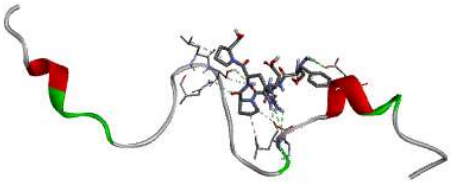

R6 : SFNEPHP

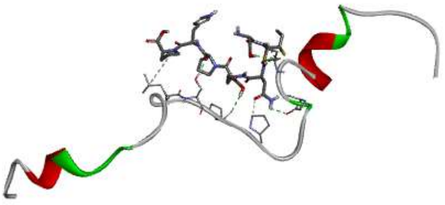

R7 : SINEPHP

b. Redundant (R) peptides.

S5 Fig. Docking structures of SR50 and Redundant (R) sets with 2LY4.B.

a. SR50: SR12.1 and SR12.2. b. Representatives of Redundant (R) peptides: R0-R3, R6 and R7.
