# Supplementary material for: Phage libraries screening on P53: Yield improvement by zinc and a new parasites-integrating analysis
Source: PLoS One. 2024 Oct 3;19(10):e0297338. doi: 10.1371/journal.pone.0297338 (PMC11449285; doi:10.1371/journal.pone.0297338)
Supplement: S8 Fig — Representative peptides are 12.1, 12.2 and 12.6. (PDF) [file pone.0297338.s009.pdf]

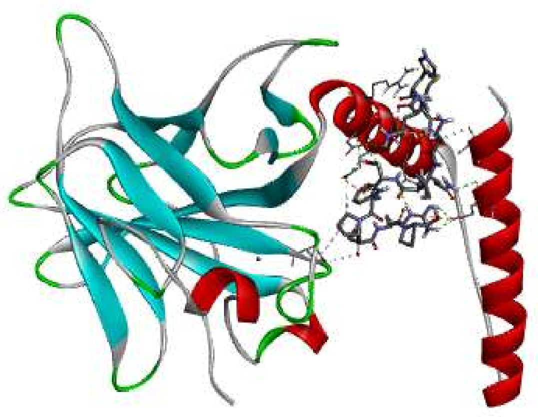

12.1 : NHMNQISFPSRP

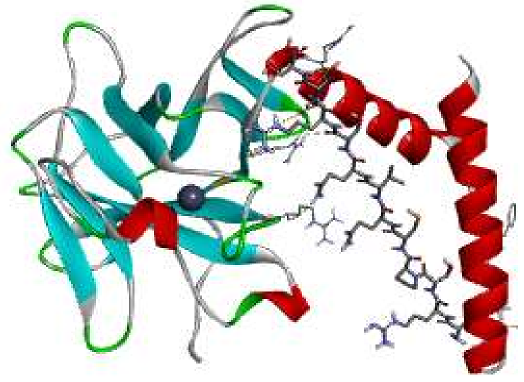

12.2 : ARSPCQVQSRTS

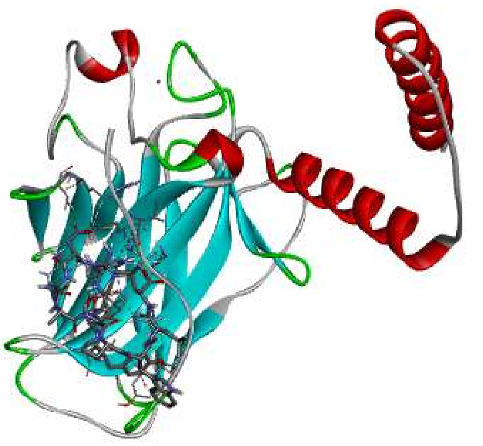

12.6:YSTHDNARPWLL

S8 Fig. Docking structures of 12-mer *non zinc* set with 3Q01 (ribbon). Representative peptides are 12.1, 12.2 and 12.6.
