# Supplementary material for: Phage libraries screening on P53: Yield improvement by zinc and a new parasites-integrating analysis
Source: PLoS One. 2024 Oct 3;19(10):e0297338. doi: 10.1371/journal.pone.0297338 (PMC11449285; doi:10.1371/journal.pone.0297338)
Supplement: S9 Fig — Peptides are 12.1–12.6. (PDF) [file pone.0297338.s010.pdf]

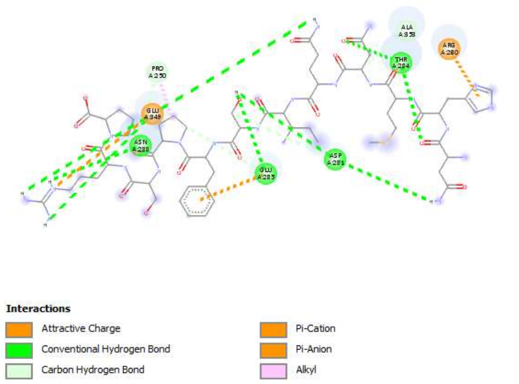

12.1: NHMNQISFPSRP

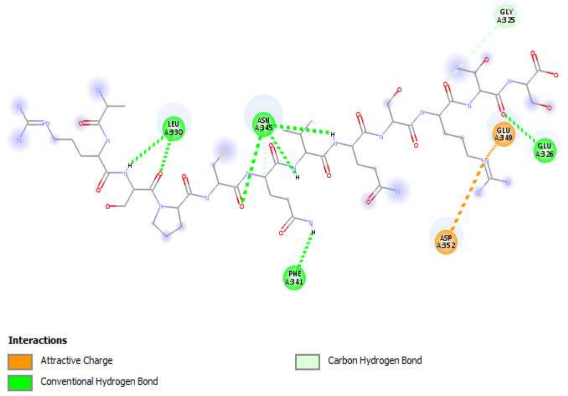

12.2: ARSPCQVQSRTS

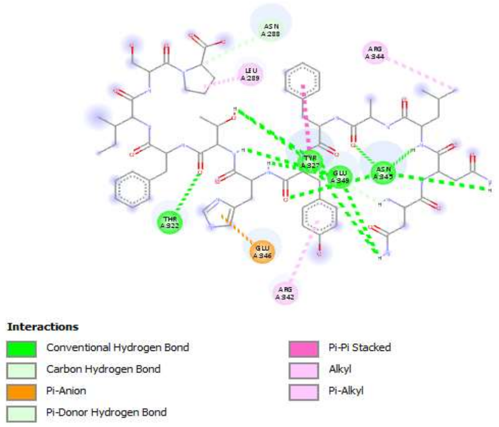

12.3: NNLAFYHTFISP

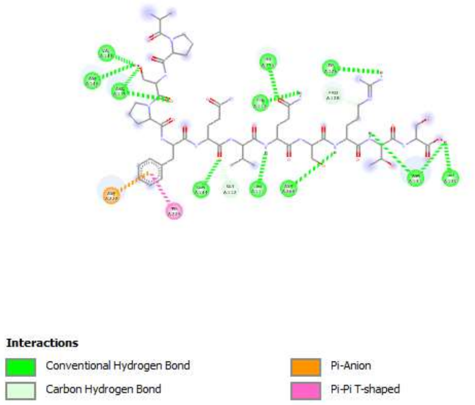

12.4: APSPFQVQSRTS

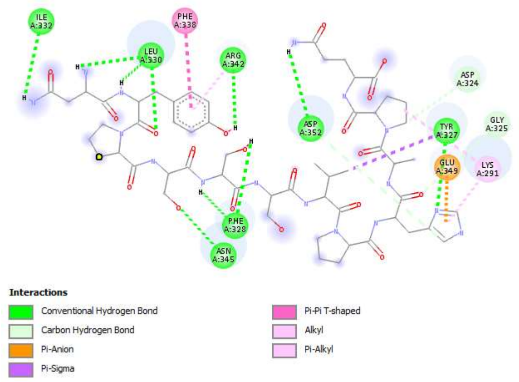

12.5: NYPSSSVPHAPQ

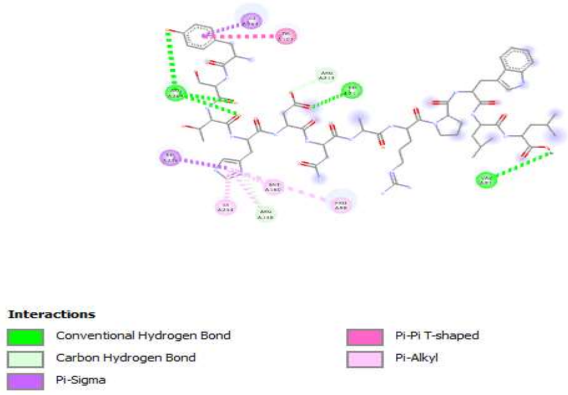

12.6:YSTHDNARPWLL

S9 Fig. Docking structures of 12-mer *non zinc* set with 3Q01 (interactions). Peptides are 12.1-12.6.
