# Supplementary material for: Phage libraries screening on P53: Yield improvement by zinc and a new parasites-integrating analysis
Source: PLoS One. 2024 Oct 3;19(10):e0297338. doi: 10.1371/journal.pone.0297338 (PMC11449285; doi:10.1371/journal.pone.0297338)
Supplement: S10 Fig — Representative peptides are 12Z1 and 1Z3-12Z6. (PDF) [file pone.0297338.s011.pdf]

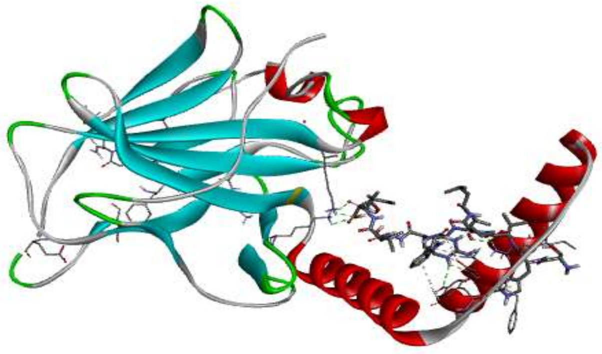

12Z1:SHVPLARWSVIT

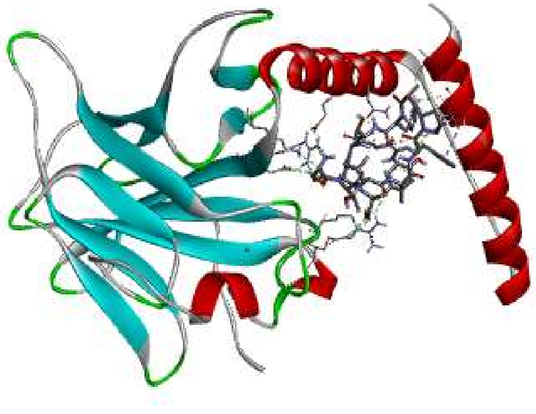

12Z3:STLVFPAHTRDY

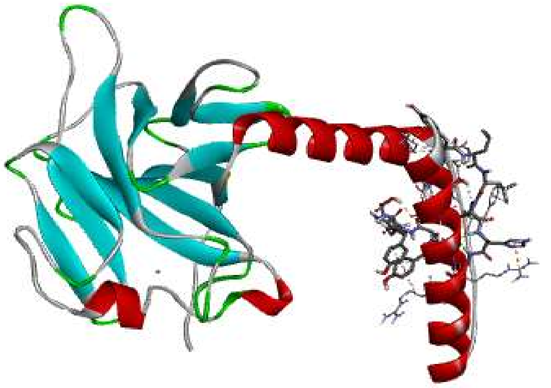

12Z4:TYLLPHSYPWYG

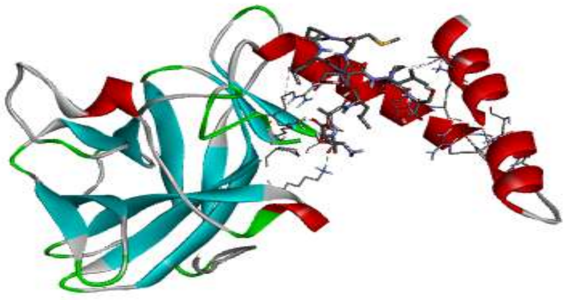

12Z5:TATLDMPLSLPS

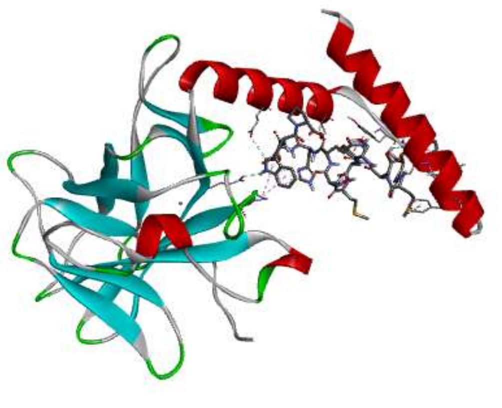

12Z6:WMDSYMSQHDWP

S10 Fig. Docking structures of 12-mer *with zinc* set with 3Q01 (ribbon). Representative peptides are 12Z1 and 12Z3-12Z6.
