# Supplementary material for: Phage libraries screening on P53: Yield improvement by zinc and a new parasites-integrating analysis
Source: PLoS One. 2024 Oct 3;19(10):e0297338. doi: 10.1371/journal.pone.0297338 (PMC11449285; doi:10.1371/journal.pone.0297338)
Supplement: S11 Fig — Representative peptides are 12Z1-12Z3 and 12Z6. (PDF) [file pone.0297338.s012.pdf]

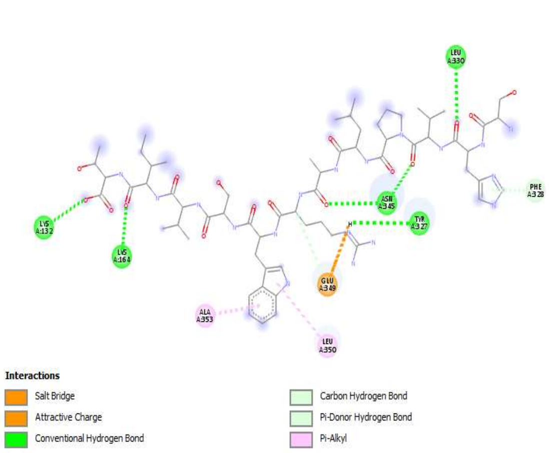

12Z1: SHVPLARWSVIT

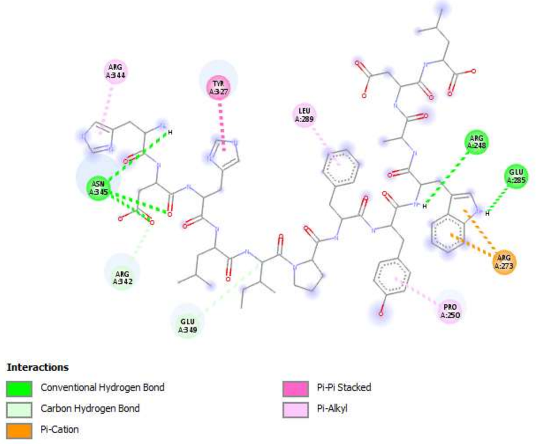

12Z2: HDHLIPFYWADL

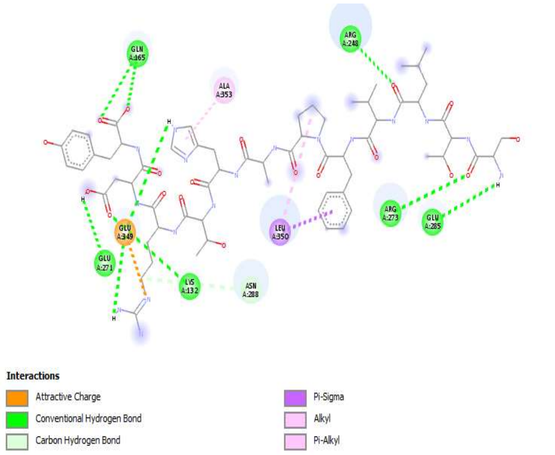

12Z3: STLVFPAHTRDY

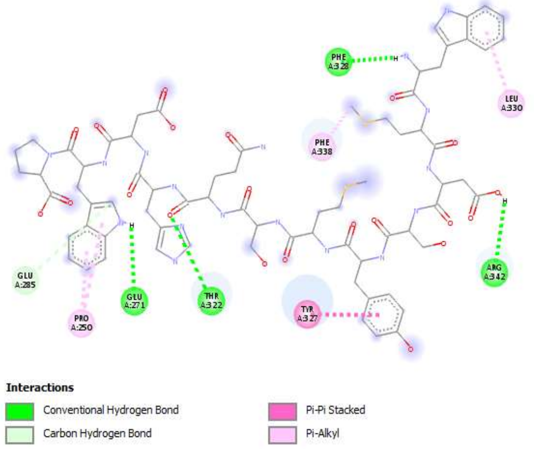

12Z6: WMDSYMSQHDWP

S11 Fig. Docking structures of 12-mer *with zinc* set with 3Q01 (interactions). Representative peptides are 12Z1-12Z3 and 12Z6.
