# Supplementary material for: Phage libraries screening on P53: Yield improvement by zinc and a new parasites-integrating analysis
Source: PLoS One. 2024 Oct 3;19(10):e0297338. doi: 10.1371/journal.pone.0297338 (PMC11449285; doi:10.1371/journal.pone.0297338)
Supplement: S12 Fig — Peptides are PD1-PD7. (PDF) [file pone.0297338.s013.pdf]

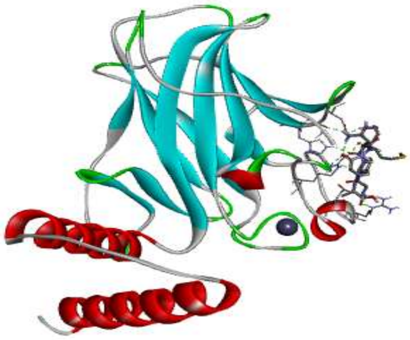

PD1 : GANMKYA

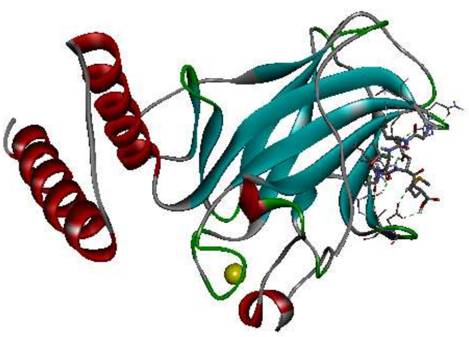

PD2: GLTATNM

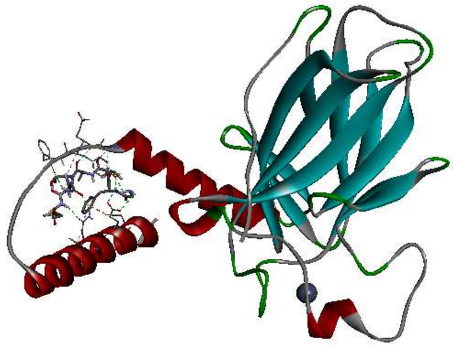

PD3: GFTATNM

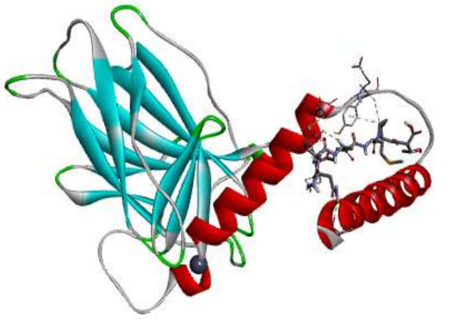

PD4 : NDAEMPT

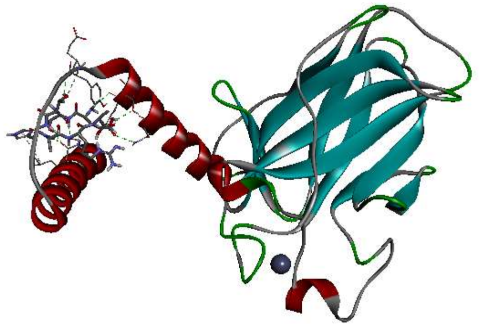

PD5: ETTHARA

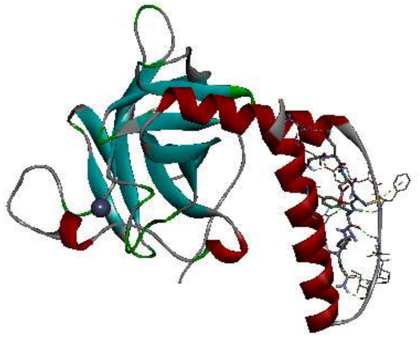

PD6: GLDCYKQ

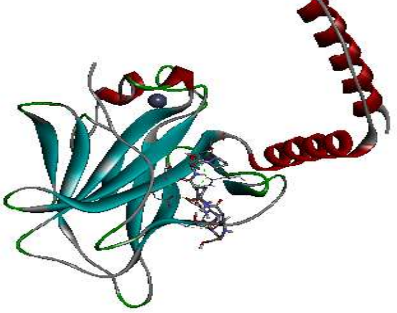

PD7: STQARTP

**S12 Fig. Docking structures of PD74 set (control) with 3Q01 (ribbon).** Peptides are PD1-PD7.
