# Supplementary material for: Phage libraries screening on P53: Yield improvement by zinc and a new parasites-integrating analysis
Source: PLoS One. 2024 Oct 3;19(10):e0297338. doi: 10.1371/journal.pone.0297338 (PMC11449285; doi:10.1371/journal.pone.0297338)
Supplement: S13 Fig — Peptides SR12.1 and SR12.2. Once ribbon (top) and interactions (down). (PDF) [file pone.0297338.s014.pdf]

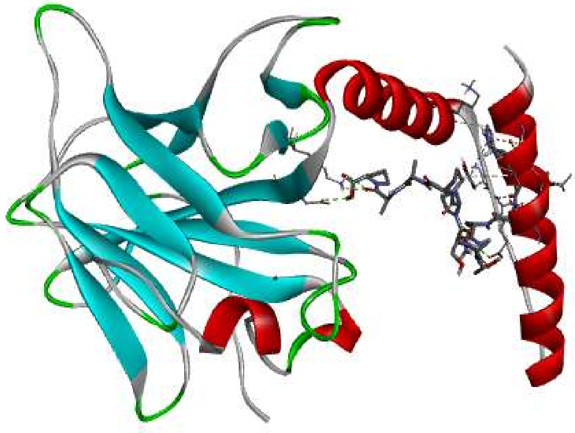

SR12.1: HLAQTASPPAAP

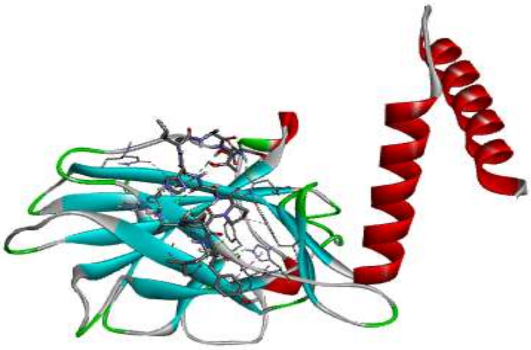

SR12.2:APLYSPSHLATS

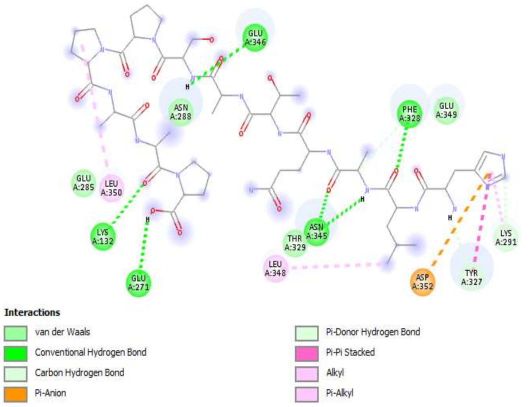

SR12.1: HLAQTASPPAAP

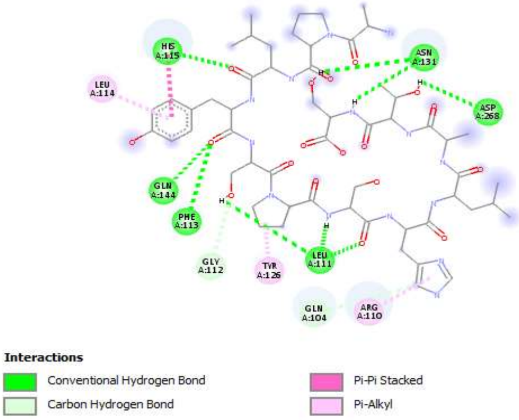

SR12.2:APLYSPSHLATS

S13 Fig. Docking structures of SR50 set with 3Q01. Peptides SR12.1 and SR12.2. Once ribbon (top) and once interactions (down).
