# Supplementary material for: Phage libraries screening on P53: Yield improvement by zinc and a new parasites-integrating analysis
Source: PLoS One. 2024 Oct 3;19(10):e0297338. doi: 10.1371/journal.pone.0297338 (PMC11449285; doi:10.1371/journal.pone.0297338)
Supplement: S15 Fig — Peptides are R2-R5 and R11-R12. (PDF) [file pone.0297338.s016.pdf]

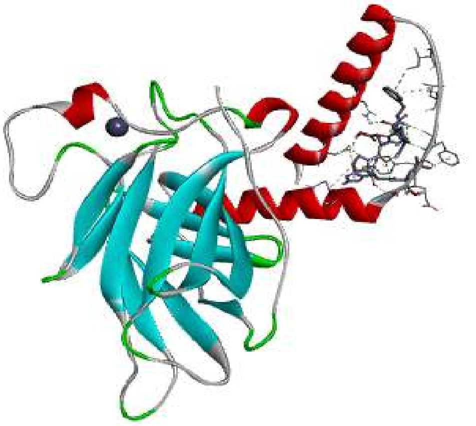

R2: PFNEPHL

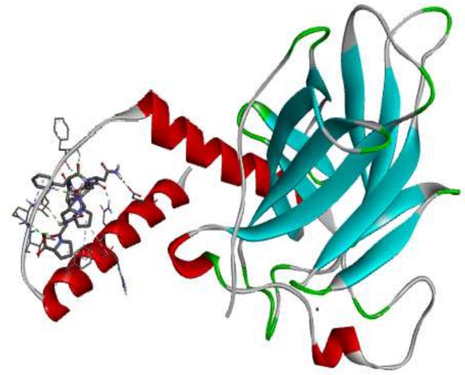

R3: PFNEPHP

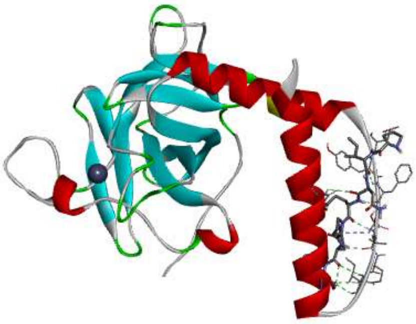

R4: PINEPHP

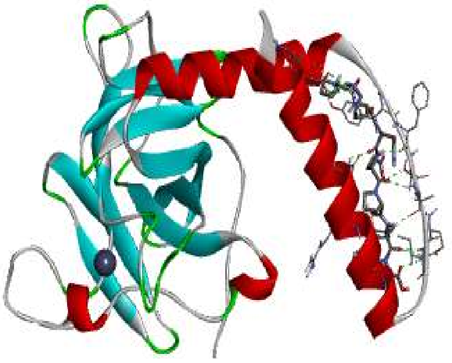

R5: PKNEPHP

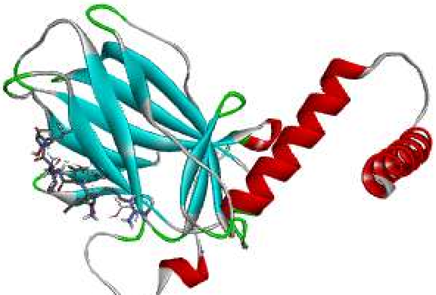

R11: LFNERHP

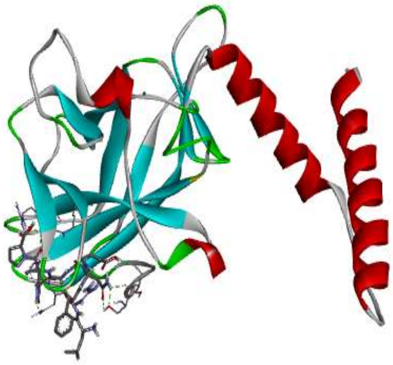

R12: PINEPHL

S15 Fig. Docking structures of Redundant set (R) Motif 2 with 3Q01 (ribbon). Peptides are R2-R5 and R11-R12.
