# Supplementary material for: Phage libraries screening on P53: Yield improvement by zinc and a new parasites-integrating analysis
Source: PLoS One. 2024 Oct 3;19(10):e0297338. doi: 10.1371/journal.pone.0297338 (PMC11449285; doi:10.1371/journal.pone.0297338)
Supplement: S16 Fig — Peptides are R13-R17. (PDF) [file pone.0297338.s017.pdf]

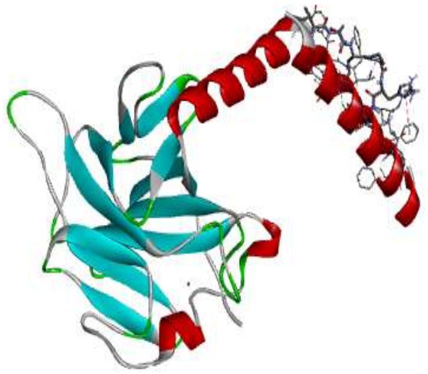

R13 : AFNEPHP

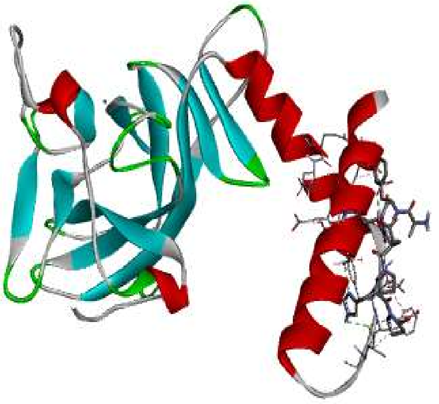

R14:AINEPHP

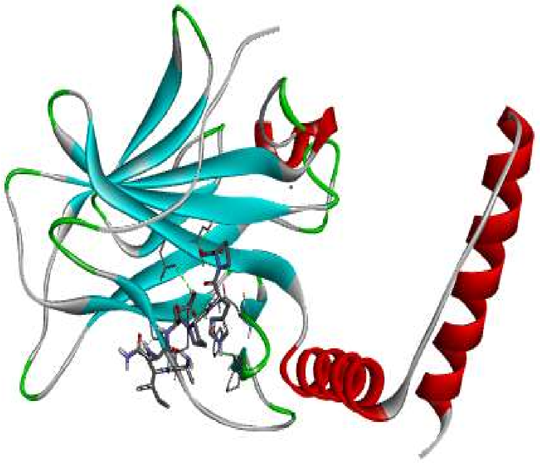

R15: AINEPHL

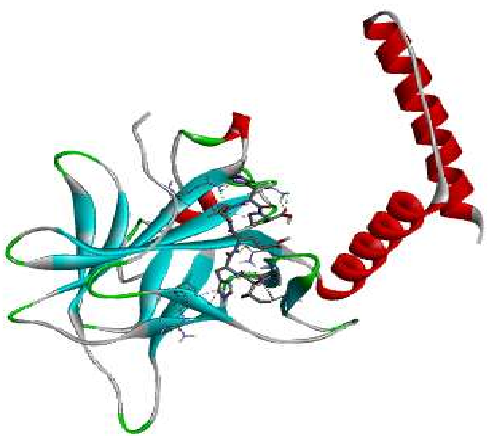

R16:AFHEPHP

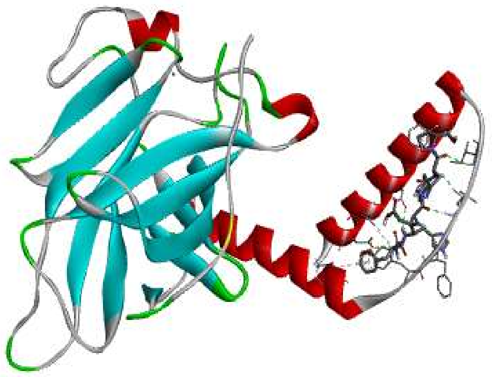

R17:AIHEPHP

S16 Fig. Docking structures of Redundant set (R) Motif 2 with 3Q01 (ribbon)(continuation).

Peptides are R13-R17.
