# Supplementary material for: Phage libraries screening on P53: Yield improvement by zinc and a new parasites-integrating analysis
Source: PLoS One. 2024 Oct 3;19(10):e0297338. doi: 10.1371/journal.pone.0297338 (PMC11449285; doi:10.1371/journal.pone.0297338)
Supplement: S17 Fig — Representative peptides are R0, R1 and R8-R10. (PDF) [file pone.0297338.s018.pdf]

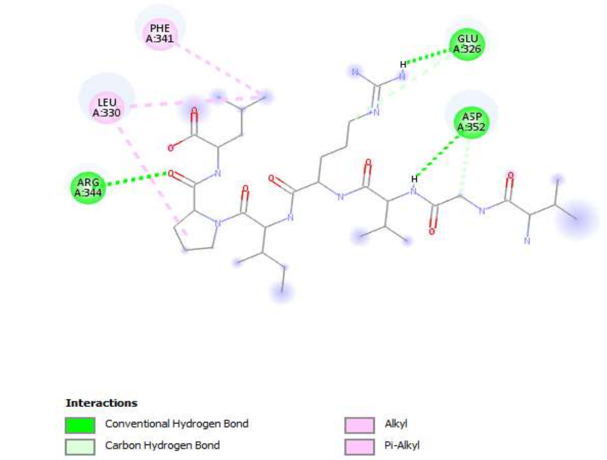

R0: VGVRIPL

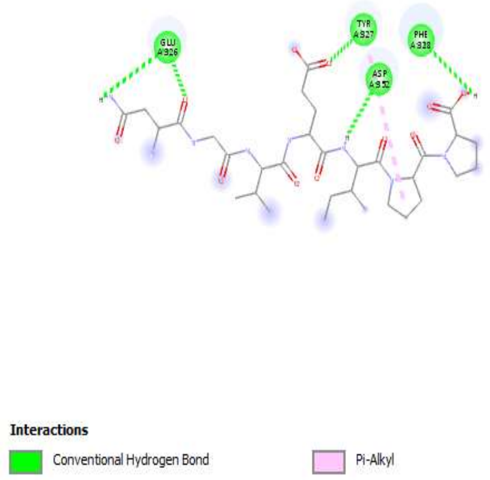

R1: NGVEIPP

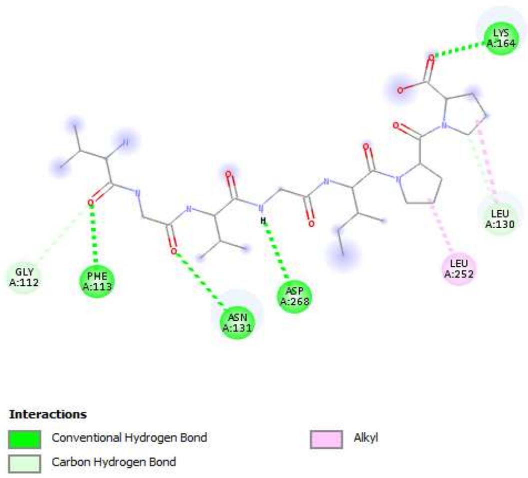

R8:VGVGIPP

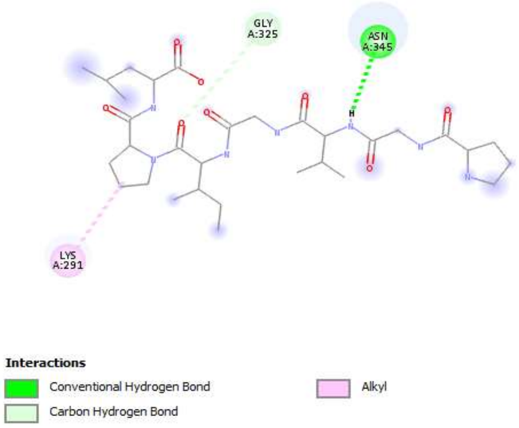

R9: PGVGIPL

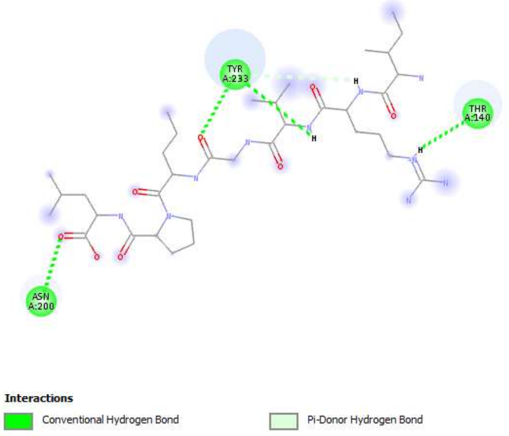

R10: IRVGIPL

S17 Fig. Docking structures of Redundant set (R) Motif 1 with 3Q01 (interactions).

Representative peptides are R0, R1 and R8-R10.
