# Supplementary material for: Phage libraries screening on P53: Yield improvement by zinc and a new parasites-integrating analysis
Source: PLoS One. 2024 Oct 3;19(10):e0297338. doi: 10.1371/journal.pone.0297338 (PMC11449285; doi:10.1371/journal.pone.0297338)
Supplement: S18 Fig — Peptides are R3, R4 and R11-R13. (PDF) [file pone.0297338.s019.pdf]

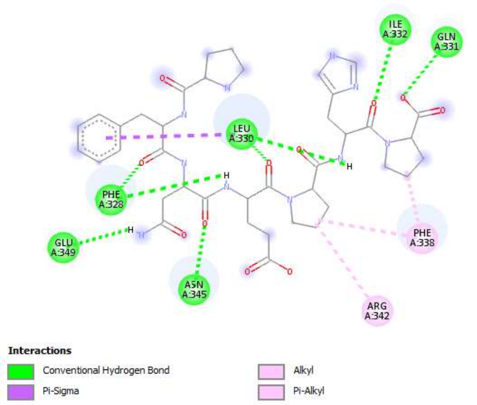

R3: PFNEPHP

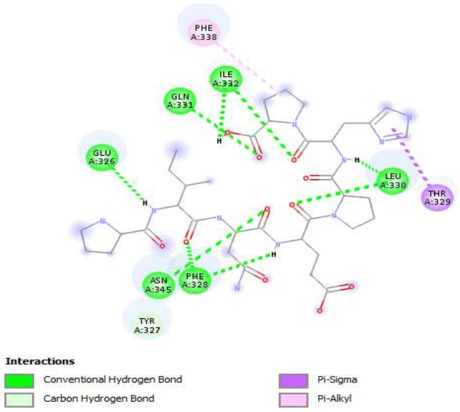

R4: PINEPHP

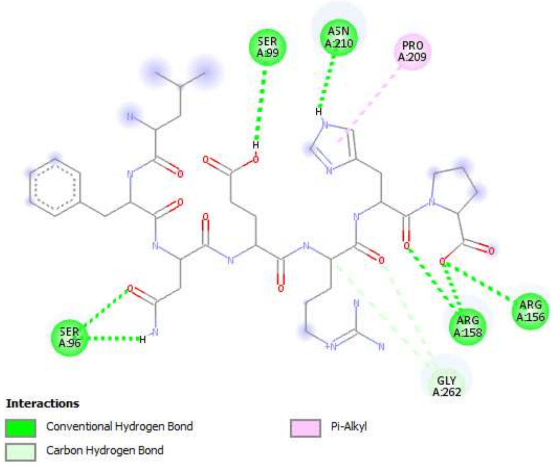

R11: LFNERHP

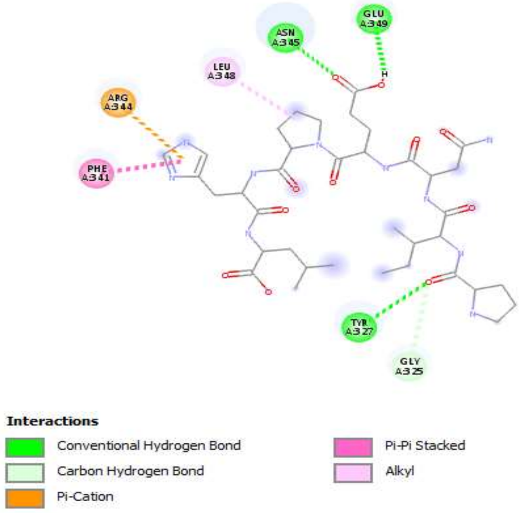

R12: PINEPHL

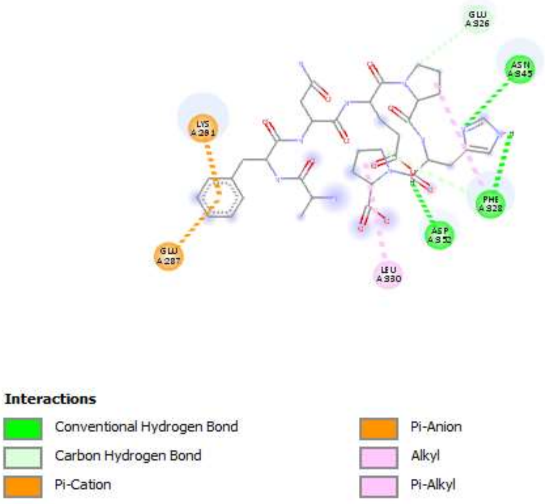

R13 : AFNEPHP

S18 Fig. Docking structures of Redundant set (R) Motif 2 with 3Q01 (interactions). Peptides are R3, R4 and R11-R13.
