# Supplementary material for: Phage libraries screening on P53: Yield improvement by zinc and a new parasites-integrating analysis
Source: PLoS One. 2024 Oct 3;19(10):e0297338. doi: 10.1371/journal.pone.0297338 (PMC11449285; doi:10.1371/journal.pone.0297338)
Supplement: S20 Fig — a. including redundant R residues. b. subtracting R residues. (PDF) [file pone.0297338.s021.pdf]

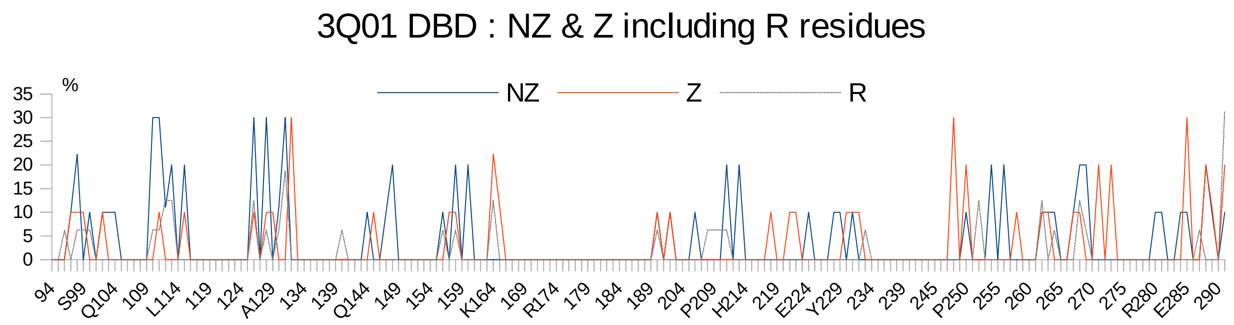

a

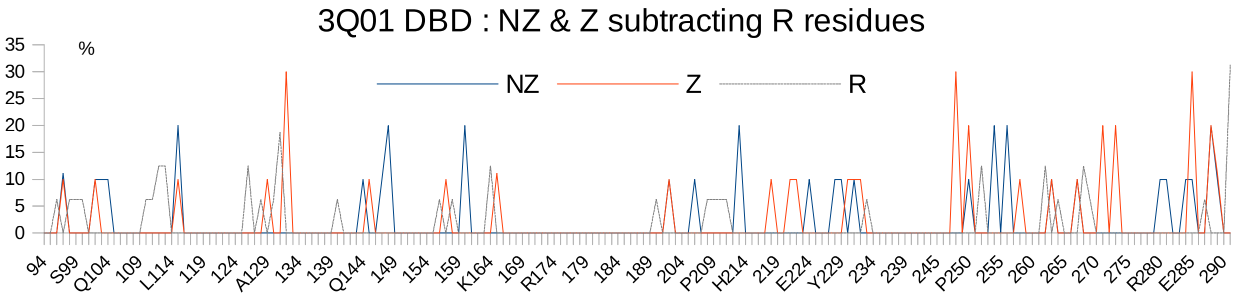

b

S20 Fig. Docking profiles of « non zinc » and « with zinc » sets on DBD region of 3Q01 structures. a. including redundant R residues. b. subtracting R residues.
