# Supplementary material for: Phage libraries screening on P53: Yield improvement by zinc and a new parasites-integrating analysis
Source: PLoS One. 2024 Oct 3;19(10):e0297338. doi: 10.1371/journal.pone.0297338 (PMC11449285; doi:10.1371/journal.pone.0297338)
Supplement: S21 Fig — a) Photo of 96-well plate after revelation and its lay-out aside. b) Histogram representing the responses of different tested phage clones. Wells are coated with protein P53. Phages are added separately. Following incubation with phages, anti-M13 antibody-HRP (Cytiva Cat# 27942101, RRID:AB_2616587) is added and response is revealed with HRP substrate (ABTS). Phage clones from phage display experiment against p53-derived peptides are respectively PD1 and PD7 for PD74 (12–61) and SR12.2 for SR50 (241–291).The 96-well plate columns were alternatively coated by protein P53 target (P) or only blocking buffer (C for Control). Each phage is essayed simultaneously on control and protein wells. A percentage of relative signals calculated as: [100-C/P*100]. Clones are ranked based on this percentage value representing the binding force. At secondary y axis: energies of docking on both 2LY4.B and 3Q01 structures. This ELISA is Representative. (PDF) [file pone.0297338.s022.pdf]

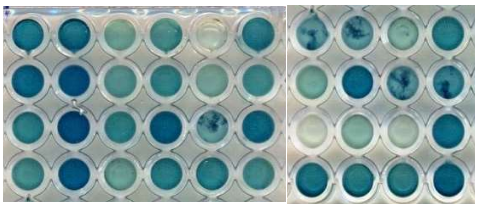

C	P	C	P	C	P	C	P	C	P
7.4	7.4	7Z3	7Z3	12Z4	12Z4	12Z1	12Z1	12.1	12.1
PD1	PD1	7Z1	7Z1	12.6	12.6	7.2	7.2	12Z2	12Z2
12Z5	12Z5	12.3	12.3	7Z4	7Z4	7.3	7.3	12.4	12.4
S12.2	S12.2	PD7	PD7	12Z3	12Z3	12.5	12.5	7.1	7.1

a

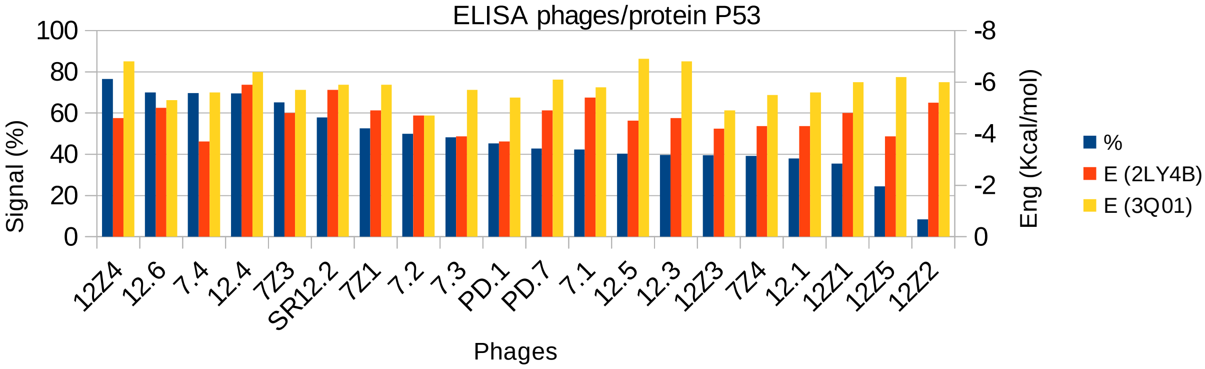

b

S21 Fig. ELISA of phages on protein P53.

a) Photo of 96-well plate after revelation and its lay-out aside. **b)** Histogram representing the responses of different tested phage clones. Wells are coated with protein P53. Phages are added separately. Following incubation with phages, anti-M13 antibody-HRP (Cytiva Cat# 27942101, RRID:AB_2616587) is added and response is revealed with HRP substrate (ABTS). Phage clones from phage display experiment against p53-derived peptides are respectively PD1 and PD7 for PD74 (12-61) and SR12.2 for SR50 (241-291).The 96-well plate columns were alternatively coated by protein P53 target (P) or only blocking buffer (C for Control). Each phage is essayed simultaneously on control and protein wells. A percentage of relative signals calculated as: [100-C/P*100]. Clones are ranked based on this percentage value representing the binding force. At secondary y axis: energies of docking on both 2LY4.B and 3Q01 structures. This ELISA is representative.
