# Supplementary material for: Phage libraries screening on P53: Yield improvement by zinc and a new parasites-integrating analysis
Source: PLoS One. 2024 Oct 3;19(10):e0297338. doi: 10.1371/journal.pone.0297338 (PMC11449285; doi:10.1371/journal.pone.0297338)
Supplement: S22 Fig — Docking points of Redundant peptides are subtracted from the other sets to show only the “specific interactions”. At left, are presented docking profiles to 2LY4.B and at right are presented docking profiles to 3Q01. (a,b) : 7 Non Zinc, (c,d) : 7 With Zinc, (e,f) : 12 Non Zinc, (g,h) : 12 With Zinc, (i,j) : PD74 recognizing set, (k,l) : SR50 recognizing set and (m,n) : Redundant (R) set. (PDF) [file pone.0297338.s023.pdf]

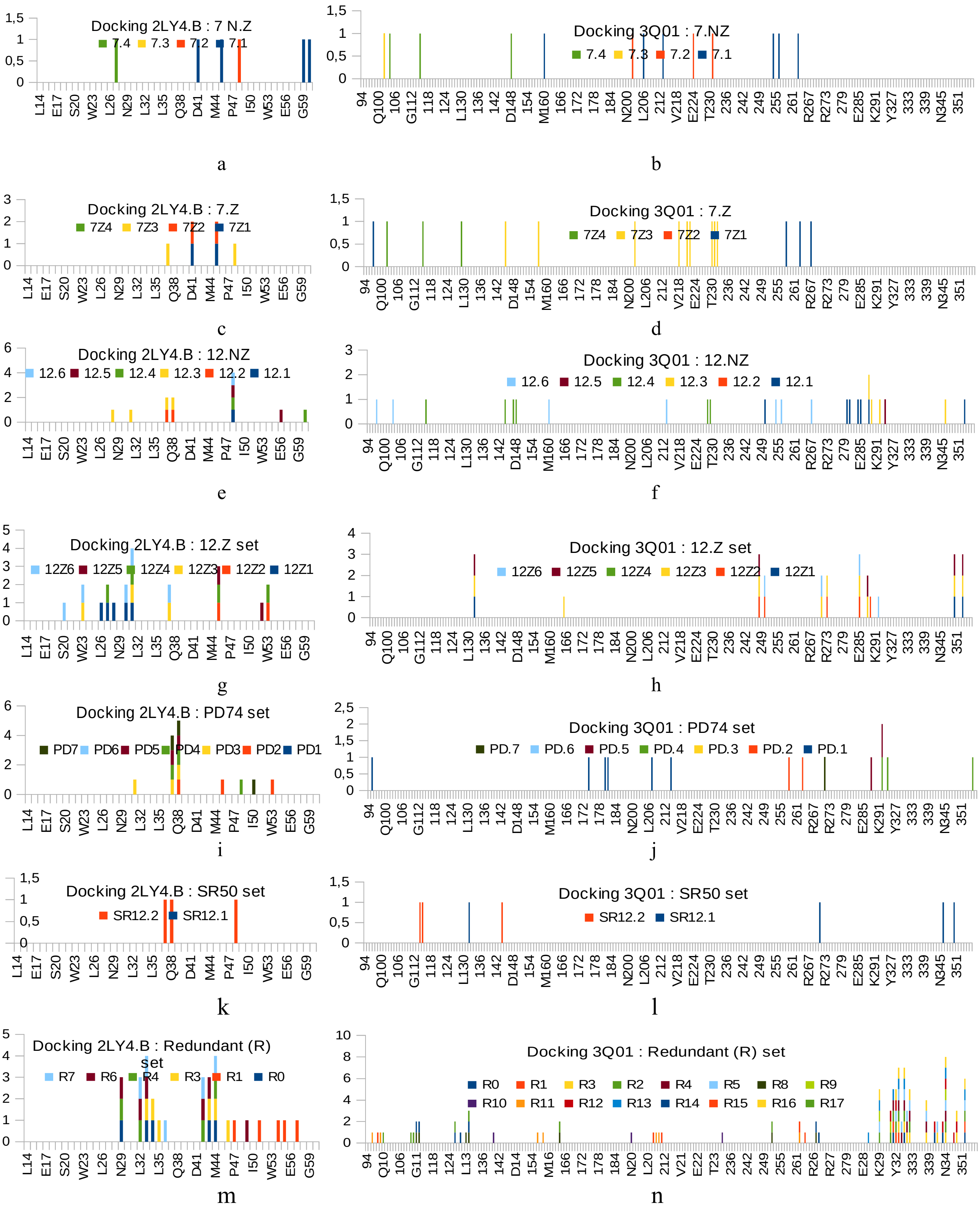

S22 Fig. Docking profiles of individual peptides.

Docking points of Redundant peptides are subtracted from the other sets to show only the “specific interactions”. At left, are presented docking profiles to 2LY4.B and at right are presented docking profiles to 3Q01. (a,b) : 7 Non Zinc, (c,d) : 7 With Zinc, (e,f) : 12 Non Zinc, (g,h) : 12 With Zinc, (i,j) : PD74 recognizing set, (k,l) : SR50 recognizing set and (m,n) : Redundant (R) set.
