# Supplementary material for: Phage libraries screening on P53: Yield improvement by zinc and a new parasites-integrating analysis
Source: PLoS One. 2024 Oct 3;19(10):e0297338. doi: 10.1371/journal.pone.0297338 (PMC11449285; doi:10.1371/journal.pone.0297338)
Supplement: S1 File — a) Individual peptides docking on 2LY4.Chain B. b) Analysis of retained docking interactions in 3Q01. (PDF) [file pone.0297338.s024.pdf]

S1 File. Analysis of docking profiles of individual peptides.

a) Individual peptides docking on 2LY4.Chain B

7-mer set of P53-recognizing peptides (S1 Fig):

Peptide 7.1: HTWLRSA shows an interesting docking, making a loop between region (41-47) and the end (57-60). It is the single motif that attaches to this end (57-60). 7Z1: WSWPRFL and 7Z2: MQAPSPM dock at the same place (41-54) and almost the same residues. Structurally, this makes a loop spanning helix (46-51) and connecting the region before it to the C-terminus. However, a part of the binding (P47/F54) is ignored if we correct by subtracting the R residues. The *remaining bindings are: (D41-H1/W3, L45-H1, G59-R5 and P60-L4/R5) for 7.1, (D41-W3/R5 and L45-W1/S2) for 7Z1 and (D41-Q2 and L45-Q2) for 7Z2.*

"12 non-zinc" set (S2 Fig):

Peptide 12.3: NNLAFYHTFISP docks in the region (28-38) but two positions remain after the double correction (subtraction of *R* and *SR50*), E28 (N1) and V31 (A4). Additionally, 12.4:APSPFQVQSRTS, 12.5: NYPSSSVPHAPQ and 12.6: YSTHDNARPWLL dock to similar regions and in the same way making a loop spanning the (46-51) helix and connecting the front region to the C-terminus. However, the correction leaves only 12.4 and 12.5 with a P60 (P4) and E56 (Y2) positions respectively.

"12 with zinc" set (S3 Fig):

Peptides 12Z6: WMDSYMSQHDWP (20-37), 12Z3: STLVFPAHTRDY (23-43) and 12Z1: SHVPLARWSVIT (26-35) dock with the indicated similar regions in the N-terminal part. The *peptide 12Z6 shows an interesting docking:* a close link in particular *with residues S20 (W11), W23 (Y5, W11), N30 (P12) and V31 (W1, P12) which are retained*, linked by the peptide residues indicated. 12Z1 has a rather delimited (strong) docking region distant from any secondary structure including that at the N-terminal extremity (14-20). The *L26P27E28-N30V31 region is retained after*

correction. The binding is as follows: L26-P4, P27-P4, E28-W8/S9, N30-T12 and V31-H2. 12Z3 retains two positions: W23 (Y12) and V31 (Y12).

Otherwise, 12Z4: TYLLPHSYPWYG (27-35 and44-54), 12Z2: HDHLIPFYWADL (29-36 and 43-53) then 12Z5: TATLDMPLSLPS (34_36 and 45_52) dock with the large regions indicated. These regions are more close to the C-terminus, spanning the second red/green structure (46-51). The 12Z2 and 12Z4 peptides have more spatial and three-dimensional docking than the 12Z5 due to their sequences. They contain Imidazole, Proline and aromatic radicals and residues. However, after correction by *R* residues subtraction, only 12Z4 saves its spread docking with four positions: P27 (T1), V31 (Y2), L45 (P9) and W53 (Y8). This brings out the pattern T1Y2-Y8P9 as relevant. 12Z2 joins 12Z5 by linking only two positions: L45 and Q52(12Z5)/W53(12Z2). The binding is composed of: (L45-P6 and W53-W9) for 12Z2 and (L45-L4 and Q52-T1) for 12Z5.

PD74 set (S4 Fig):

Regarding this group of peptides, they all dock with a region in the (32_50). And some of them interact with the helix (46-51): PD1 (GANMKYA), PD2 (GLTATNM), PD4 (NDAEMPT), PD3 (GFTATNM) and PD7 (STQARTP). PD5 (ETTHARA) has a concentrated docking located in a small region (34_44). PD6 (GLDCYKQ) seems to have fewer bonds than the others: 2 liaisons with 2 residues, P36 and L43. The remaining interactions after eliminating redundancy (and non-specificity) are: (L45-L2 and W53-G1/L2) for PD2, (V31-F2) for PD3, (I50-P7) for PD7. Most of the interactions of this set therefore seem non specific almost like the ubiquitous *R* set.

R and SR50 sets (S5 Fig):

The binding of the R set (6 representative motifs) remains the same since no corrections have been made. Only R1 (NGVEIPP) has a bond shifted to the right (C-ter end) (P47-E51-F54-T55-D57).

The rest of the group docks as follows: (N29-S33-P34-L43-M44) for R0, (S33-P34-L35-L43-M44-S46) for R3, (N29-L32-D42-M44) for R4, (N29-L32-S33-D42-L43-D49) for R6 and (L32-S33-P36-D42-M44) for R7.

This set reaches helix (42-49). Regarding the control docking of *SR50* set, only SR12.2 has a remaining bond itself considered as non-specific: (S37:S7-Q38:S7/H8-D48:H8/T11/S12).

b) Analysis of retained docking interactions in 3Q01

Some peptides are simply rejected after R correction, we cite: 7Z2, 12.2, 12Z4 although other interactions can still be rejected since they are found with the PD74 or SR50 sets (outside the 241-291 region); 12.3 and 12.5 can therefore also be ignored.

Individually for each peptide, as shown by **(Fig 2)**, we have:

7.1 retains: M160-H1, L206-L4, R213-W3, I254-H1 and T256-H1. 7.2 retains: R202-S4, E224-L1/Q5, T231-N3. 7.3 retains only T102-S4. 7.4 retains: Q104-R7 and D148-R7. 7Z1 retains: V97-W3 and R267-W3. 7Z3 (AAAFTQS) retains a good number and distribution of interactions: L145-F4, V157-F4, R202-T5, V218-F4, E221-S7, P222-S7, T230-F4, T231-A2 and I232-F4. Obviously the F4 of this peptide is highly interactive; with essentially hydrophobic bonds. 7Z4 retains: T102-E3 and A129-T2.

For 12.4, are retained: V147- S3, D148-S3, D228-F5 and Y229-F5. For 12.1: R280-H2, D281-N1/S7, T284-N1/N4, E285:OE2-S7/F8/P9, P250-P9 and A353-N4. For 12.6: V97-L12, M160-H4, R213-D5, I254-H4, T256-H4 and R267-Y1/S2/T3. 12Z1 retains one position: A353-W8. 12Z2 retains all its interactions: R248-W9, P250-Y8, R273-A10, E285-A10 and L289-F7. 12Z3 retains: Q165-Y12, R248-L3, R273-S1, N285-S1 and A353-H8. 12Z5 retains: R248-L8, A353-P11. 12Z6 retains: Pro250-W11 and E285-W11.

We assemble the retained residues in peptides for all these sets, resulting in: for the 7-mer set: 7.1 (H1W3L4), 7.2 (L1N3S4Q5), 7.3 (S4), 7.4 (R7), 7Z1 (W3), 7Z3 (A2F4T5S7) and 7Z4 (T2E3). For 12-mer motifs: 12.1 (N1H2N4S7F8P9), 12.4 (S3F5), 12.6 (Y1S2T3H4D5), 12Z1 (W8), 12Z2 (F7Y8W9A10), 12Z3 (S1L3H8Y12), 12Z5 (L8P11) and 12Z6 (W11).
